# Clinical Characteristics of *Alternaria* Keratitis

**DOI:** 10.1155/2014/536985

**Published:** 2014-03-20

**Authors:** Ching-Hsi Hsiao, Lung-Kun Yeh, Hung-Chi Chen, Hsin-Chiung Lin, Phil Y. F. Chen, David H. K. Ma, Hsin-Yuan Tan

**Affiliations:** ^1^Department of Ophthalmology, Chang Gung Memorial Hospital, No. 5 Fu-Hsin Road, Kweishan 333, Taoyuan, Taiwan; ^2^College of Medicine, Chang Gung University, Taoyuan, Taiwan

## Abstract

*Purpose*. *Alternaria* spp. are an uncommon cause of mycotic keratitis. Previous studies on *Alternaria* keratitis have generally been limited to case reports. We examined the clinical characteristics of *Alternaria* keratitis in this study. *Methods.* The characteristics and outcomes of 7 patients with culture-proven *Alternaria* keratitis treated in our hospital were compared with 25 previously reported cases. *Results.* The risk factors for *Alternaria* keratitis were trauma in 5 patients and soft contact lenses in 1 patient. Six patients with early diagnosis (<2 weeks) were cured with medical antimicrobial treatment; a patch graft was required in 1 patient with perforation. When incorporated with previous reports on *Alternaria* keratitis (*n* = 32), 14 (44%) infections followed trauma, 10 (31%) were associated with preexisting corneal disease or previous ocular surgery, and 5 (16%) occurred in soft contact lens wearers. Successful medical treatment was achieved in 23 (72%) patients, including 10 out of 21 eyes (48%) treated with natamycin and/or amphotericin B. Therapeutic penetrating keratoplasty was performed in 9 (28%) cases. *Conclusions*. *Alternaria* keratitis is generally associated with specific risk factors and responds to medical treatment when early diagnosis is performed and prompt antifungal treatment is initiated.

## 1. Introduction

*Alternaria *is a filamentous fungus from the dematiaceous family, a group of darkly pigmented molds that are ubiquitous in soil, plants, food, and indoor air environments [1]. This fungus can cause opportunistic human infections, including cutaneous and subcutaneous infections (74.3%), oculomycosis (9.5%), invasive and noninvasive rhinosinusitis (8.1%), and onychomycosis (8.1%) [1]. Oculomycosis caused by* Alternaria* is primarily keratitis in patients with immunocompromised ocular surface, previous surgery, and trauma [2–23]. Previous experience with* Alternaria* keratitis has ranged variably from penetrating keratoplasty to medical cure, but with a variable response to a variety of topical and systemic antifungals [2–23]. Literature concerning corneal ulcers caused by* Alternaria* consists primarily of case reports [2–23]. We performed a 10-year retrospective review of patients with culture-proven* Alternaria* keratitis in our hospital to study the clinical characteristics of* Alternaria *keratitis and compared our experience with previously reported cases.

## 2. Materials and Methods

This study followed the Declaration of Helsinki and was approved by the Institutional Research Ethics Board at Chang Gung Memorial Hospital, Taiwan (IRB102-4073B). We searched the computer database of the microbiology laboratory in our hospital and reviewed the corresponding medical records to identify patients with culture-proven* Alternaria* keratitis, who were treated between January 1, 2003, and December 31, 2012. Both inpatients and outpatients were included. The data collected included demographic information, medical and ocular history, signs and symptoms, predisposing factors, presenting and final visual acuity, treatment, and length of follow-up. Smears and cultures from corneal scrapings for bacteria, mycobacteria, and fungi were performed in all patients. With standard microbiological culture techniques, the scrapings were inoculated directly onto blood, chocolate, a modified Sabouraud agar, a Lowenstein-Jensen agar slant, and a thioglycollate broth. Microbial cultures were considered to be significant as growth of the same organism on two or more culture media or as growth on one medium of organisms seen on stained smears of corneal scrapings. Fungal identification was based on morphology.

Medical treatment was considered successful if corneal infection was resolved during antifungal therapy and did not recur after topical agents were discontinued. Previously reported cases of* Alternaria* keratitis were identified by searching the MEDLINE database and then restricting the search to corneal infections that had laboratory evidence and outcome details written in English. Demographic data, risk factors, and management information were extracted and tabulated. Characteristics of previously reported cases were compared with the current series by using the Wilcoxon rank-sum test for continuous variables and the Fisher exact test for categorical variables. A *P* value under 0.05 was considered statistically significant. All statistical analyses were performed using SPSS software, version 20 (IBM SPSS Statistics, New York, NY, USA).

## 3. Results

### 3.1. Clinical Features

*Alternaria* spp. were isolated from 7 patients (3.4%) with fungal keratitis in our hospital during the 10-year period.  Table 1 summarizes the clinical data.

**Figure 1:**
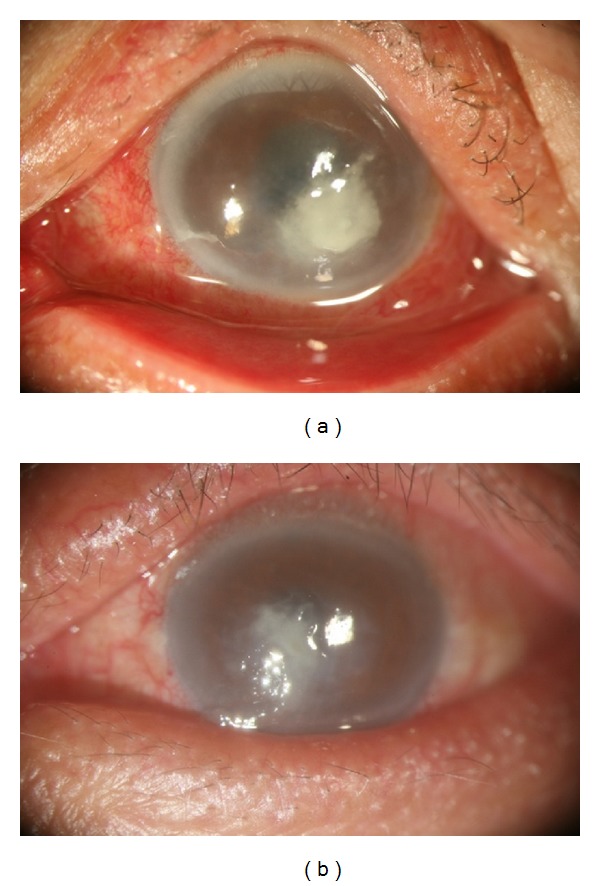
*Alternaria *keratitis presenting as a central corneal infiltrate with feathery margin. (a) Patient 3, coinfected with* Mycobacterium chelonae*. (b) Patient 5.

The median age was 62 years (range 17–76 years). The mean follow-up time was 11 months (range 2–29 months). The patients included 3 women and 4 men. Two ulcers developed in the right eye and 5 in the left eye. Predisposing factors for keratitis were identified for 6 patients; 5 infections followed corneal trauma and one was associated with soft contact lenses. One diabetic farmer (Patient 3), who underwent cataract surgery in his left eye 1 month before presentation, reported no precipitating factors. Whether Patient 4 had used corticosteroids was unclear, but the other 6 patients had no such history. All of the patients displayed similar manifestations of pain and redness in the eyes. The duration between onset of symptoms and diagnosis ranged from 4 to 10 days in 6 patients; Patient 4 was previously treated elsewhere approximately 4 months and referred to us for diagnosis. Six patients had centrally located and medium-sized (2–6 mm at its greatest dimension) corneal infiltrate with a feathery margin (Figure 1), and Patient 7 had a peripheral 1 × 1 mm ulcer.

None had a hypopyon. Topical antifungal agents, occasionally used in combination, included natamycin 5% suspension and amphotericin B 0.15% for 6 patients. The culture result of the corneal scrapings from Patient 3 revealed* Mycobacterium chelonae* in addition to* Alternaria* spp.; therefore, amikacin (25 mg/cc) and ciprofloxacin 0.3% were added later. Patient 7 exhibited a small peripheral soft contact lens-related corneal ulcer, which healed completely after treatment with amikacin (25 mg/cc) and cefazolin sodium (25 mg/cc) for 3 days. Thus, she did not receive antifungal treatment even though the culture result later revealed* Alternaria* spp. No patients received systemic antifungal therapy. All of the patients, except Patient 4, responded well to medical antimicrobial treatment. Two patients (Patients 3 and 4) underwent superficial keratectomy for debridement and promotion of the penetration of antimicrobial medication. Patient 4 eventually developed a perforated ulcer; thus a patch graft with a glycerol-preserved cornea was performed and no recrudescent infection developed. All of the patients preserved useful vision (≥20/200); the limited visual rehabilitation was primarily caused by central corneal scarring and cataracts if existed.

### 3.2. Literature Review

Twenty-five previously reported cases of* Alternaria* keratitis presented surgical or other outcome information (Table 2) [2–23]. These 17 men and 8 women ranged from 26 to 72 years of age.

Risk factors for infections are summarized in Table 3.

Nine out of twenty-five (36%) infections followed corneal trauma; 10 (40%) occurred in the eyes with prior corneal disease or surgery and 4 (16%) were associated with contact lenses. Fourteen (56%) had a definite history of corticosteroid usage before diagnosis of fungal keratitis. Topical antifungal agents, occasionally administered in combination, included natamycin, amphotericin B, miconazole, ketoconazole, voriconazole, flucytosine, fluconazole, and capofungin. Some patients also received systemic antifungal therapy, including oral itraconazole and voriconazole. Two patients received an intracameral or intrastromal injection of voriconazole. Seventeen (68%) patients were cured with medical treatment, but only 5 out of 15 patients, who received natamycin or amphotericin B, achieved successful medical treatment. Therapeutic keratoplasty was performed on 8 patients (32%) (Table 3).

When incorporated with our cases of* Alternaria* keratitis (*n* = 32), 14 (44%) infections followed trauma, 10 (31%) were associated with preexisting corneal disease or previous ocular surgery, and 5 (16%) occurred in soft contact lens wearers. A definite history of corticosteroid use was observed in 14 (44%) patients. Successful medical treatment was achieved in 23 (72%) patients, including 10 out of 21 eyes (48%) treated with natamycin and/or amphotericin B. Therapeutic penetrating keratoplasty was performed in 9 (28%) cases.

## 4. Discussion

First recognized in 1975 [2],* Alternaria *spp. are an uncommon cause of corneal infection and account for 3.3% to 8.7% of mycotic keratitis [24–28].* Alternaria* spp. were responsible for 3.4% of mycotic keratitis over the 10-year interval observed in our hospital. Previous studies of* Alternaria* keratitis have generally been limited to case reports; therefore, this may be the greatest number of cases of* Alternaria* keratitis reported at one institution. Our findings indicate that* Alternaria *keratitis was generally associated with specific risk factors, including trauma and contact lens usage, and responded well to conventional antifungal drugs.

The most common risk factors for* Alternaria* keratitis is trauma (Table 3), considering that 5 out of 7 patients had a history of trauma in our study. Surgery and preexisting corneal diseases are also commonly associated with* Alternaria* keratitis (Table 3), but none of our 6 patients with identified risk factors had such a history. The difference was possibly caused by differences in geographic regions and study populations. Soft contact lens-related fungal keratitis has increased in frequency over the past few years. An outbreak of* Fusarium* keratitis associated with the use of ReNu with MoistureLoc (Bausch & Lomb, Rochester, NY, USA) solution occurred [29]. Brooks et al. reported that* Alternaria* spp. possessed the ability to penetrate the matrix of soft contact lenses, but it was not sufficiently common to cause a contact lens-related keratitis [30]. However, recent studies have reported several cases of contact lens-related* Alternaria* keratitis [16, 19, 20], and a contact lens wearer also participated in our study. Whether contact lens-related keratitis caused by* Alternaria* increases and the relationship between* Alternaria* and various contact lens brands and disinfection solutions warrant further study.

In the present study, 5 out of 7 patients responded well to conventional topical antifungal medication such as natamycin and amphotericin B, but previous case reports on* Alternaria* keratitis have indicated variable responses to a variety of topical and systemic antifungals. Surgical interventions have occasionally been necessary (Tables 2 and 3). Limited information concerning the in vitro antifungal susceptibility of* Alternaria* spp. is available [1, 16, 26, 31]. In general, amphotericin B showed variable in vitro activity; the triazoles, including itraconazole, voriconazole, and posaconazole, presented low minimum inhibitory concentrations (MICs);* Alternaria *spp. seemed to be susceptible to terbinafine and caspofungin in vitro [1, 16, 26, 31]. However, in vitro drug susceptibility based on serum standards may not be accurate because high antimicrobial concentrations achieved with topical instillation may overcome the resistance as determined by the MICs of serum concentrations. Recently, using voriconazole for the treatment of fungal keratitis has become a trend [14]. Voriconazole possesses excellent bioavailability, and therapeutic aqueous and vitreous levels are achieved after topical and oral administration of voriconazole. Successful management of* Alternaria* keratitis clinically resistant to natamycin and amphotericin B with oral and topical voriconazole has recently been reported [12, 14, 18, 20, 21, 23]. Voriconazole appears to be a promising agent for* Alternaria *keratitis.

The infections resolved without using any antifungal medication in Patient 7, who was a contact lens wearer. A study on the outbreak of* Fusarium* keratitis associated with soft contact lenses also reported that infections in 11 eyes (16.2%), treated with topical fortified cefazolin and gentamicin, were resolved without requiring antifungal therapy [29]. All of these cases were diagnosed at an early stage, presented with small, superficial, paracentral, or peripheral lesions, similar to our case. Host immunoresponsiveness, a decrease in organism loading after corneal scrapings, and the possible antifungal effects of certain antibiotics [32] are likely associated with the solution of infection in these cases.

Two of our patients (Patients 3 and 4) underwent superficial keratectomy. Superficial keratectomy may aid in the medical management of fungal keratitis by increasing drug penetration, removing infected corneal tissue, and subsequently reducing the microbial load. However, the increase of corneal perforation after early keratectomy is concerning. Lin et al. studied treatment outcome, cost of care, and long-term complications in patients with moderate* Fusarium* keratitis who received early keratectomy compared with those treated medically and observed that the early keratectomy group had a shorter hospital stay, shorter disease duration, lower hospitalization costs, and lower rates of corneal perforation than the medical therapy group did [33]. Topical natamycin solution was prescribed for our Patient 4, having received chronic keratitis treatment elsewhere for 4 months, and superficial keratectomy was performed on the day after admission, based on a presumed diagnosis of fungal keratitis. Unfortunately, perforation occurred 5 days later; thus, a patch graft with a glycerol-preserved cornea was performed. The progressive and perforated ulcer in this patient was partially caused by inadequate medical treatment, because previous reports indicated that* Alternaria* spp. appeared to be largely resistant to natamycin clinically [12, 16]; however, in our study, 2 other patients responded well to natamycin solely. Superficial keratectomy might be at least partially responsible for perforation in this case. How to judge which cases with* Alternaria* keratitis would benefit from early superficial keratectomy is critical and requires further investigation.

Our patients appeared to respond well to medical treatment and presented more favorable outcomes when compared with previous case reports. Although some reporting bias may exist, early diagnosis (<2 weeks) and prompt instillation of antifungals may have helped to halt the infection, because most of our patients had a typical history and clinical manifestations of fungal keratitis. In addition, most of our patients did not previously use corticosteroids that may promote fungal infection.

This study is limited by its retrospective character and relatively small sample size. Variations in the treatment protocol among the various physicians were observed. In addition, isolates were not identified at the species level and no drug susceptibility was available. Moreover, the patient selection criteria may have influenced the data interpretation because our patients came from a referral-based, tertiary-care institution. Therefore, the results should be generalized with caution.

## 5. Conclusions

*Alternaria* spp. are a relatively uncommon cause of corneal infection, and this study showed that* Alternaria *keratitis was generally associated with specific risk factors and responded well to medical treatment. Accurate diagnosis and prompt treatment may preserve ocular integrity and visual acuity.

## Figures and Tables

**Table 1:** Clinical data of patients with *Alternaria* keratitis.

Patient	Risk factors	Location and size	Medical treatment	Surgery	Initial VA	Final VA	F/U (months)	Others
1	Trauma	C, 4 × 3 mm	Natamycin + Amphotericin B		CF	20/200	2	
2	Trauma	C, 2 × 2 mm	Amphotericin B		20/200	20/100	10	
3	Unknown, DM	C, 4 × 4.4 mm	Natamycin + amikacin + ciprofloxacin	Superficial keratectomy	20/200	20/70	16	(i) Cataract operation 1 month ago (ii) A farmer (iii) Coinfection with *M.chelonae *
4	Trauma	C, 3.6 × 3 mm	Natamycin	Superficial keratectomy, patch graft	CF	20/200	15	PKP + ECCE + IOL 5 months later
5	Trauma	C, 3 × 4 mm	Natamycin		CF	20/100	29	
6	Trauma	C, 2 × 2 mm	Natamycin + amphotericin B		20/400	20/100	4	
7	SCL	P, 1 × 1 mm	Cefazolin + amikacin		20/25	20/20	2	

DM: diabetes mellitus, SCL: soft contact lens, C: central, P: peripheral, CF: counting fingers, F/U: follow-up, PKP: penetrating keratoplasty, ECCE: extracapsular cataract extraction, and IOL: intraocular lens.

**Table 2:** Previously reported cases of *Alternaria* keratitis.

Reference, report year	Age/sex	Risk factor	Antifungal medication	Surgery	Others
Azar et al., 1975 [2]	53/M	HSV keratitis	Topical natamycin	PKP	
Ando and Takatori, 1987 [3]	53/F	PKP	Topical thimerosal, pimaricin, flucytosine		
Chang et al., 1994 [4]	55/M	Trauma	Topical miconazole, fluconazole	PKP	Combined endophthalmitis
Arrese et al., 1996 [5]	46/M	Systemic steroids	Itraconazole	PKP	
Daniel et al., 1997 [6]	45/M	Trauma	Topical ketoconazole		
Koc et al., 1997 [7]	82/M	ECCE	Topical fluconazole		
Ferrer et al., 2002 [8]	50/M	Trauma Systemic steroids	Topical amphotericin B, fluconazole	PKP	
Zahra et al., 2002 [9]	55/M	Trauma, DM	Topical amphotericin B		
Ferrer et al., 2003 [10]	66/M	Trauma	Topical amphotericin B, fluconazole		Combined with endophthalmitis
Verma et al., 2005 [11]	29/F	LASIK	—	PKP	Diagnosis after PKP
Ozbek et al., 2006 [12]	69/M	Trauma	Topical natamycin, amphoterin B → topical and oral voriconazole		
Barnes et al., 2007 [13]	59/M	KPro	Topical amphotericin B		
Bunya et al., 2007 [14]	69/M		Topical amphotericin B, natamycin → topical and oral voriconazole		
Kocaturk et al., 2007 [15]	46/F	LASIK	Topical amphotericin B + natamycin + oral itraconazole	PKP	PKP after resolution of infection
Tu, 2009 [16]	39/M	SCL	Topical voriconazole → oral voriconazole + topical natamycin → intrastromal injection of voriconazole + topical caspofungin		
Tu, 2009 [16]	45/M	Trauma	Topical natamycin + oral itraconazole → topical fluconazole		
Tu, 2009 [16]	70/M	COAG DM	Topical fluconazole		
Usui et al., 2009 [17]	55/M	Glaucoma	Topical fluconazole + miconazole → topical amphotericin B		
Shen et al., 2010 [18]	62/F	Trauma	Topical natamycin + oral ketoconazole → intracameral voriconazole		Combined endophthalmitis
Ursea et al., 2010 [19]	72/F	RGP lens Old PKP	Topical natamycin and amphotericin B	PKP	
Yildiz et al., 2010 [20]	41/F	SCL	Topical natamycin → topical and oral voriconazole		
Yildiz et al., 2010 [20]	26/F	SCL	Topical natamycin → topical voriconazole		
Martone et al., 2011 [21]	68/M	Trauma	Topical + oral voriconazole		
Neoh et al., 2011 [22]	67/M	Bullous keratopathy	Topical and intrastromal voriconazole + caspofungin	PKP	
Konidaris et al., 2013 [23]	66/F	PKP	Topical and oral voriconazole + topical amphotericin B	PKP	

M: male, F: female, HSV: herpes simplex virus, PKP: penetrating keratoplasty, ECCE: extracapsular cataract extraction, DM: diabetes mellitus, LASIK: laser-assisted in situ keratomileusis, Kpro: keratoprosthesis, SCL: soft contact lens, COAG: chronic open angle glaucoma, and RGP: rigid gas permeable lens.

**Table 3:** Clinical features of *Alternaria* keratitis.

Characteristics	Previous cases (*n* = 25)	Current cases (*n* = 7)	*P* value
Age, median year (range)	56 (26 to 72)	62 (17 to 76)	0.21
Gender, number of males (%)	17 (68%)	4 (57%)	0.32
Risk factor, number (%)			
Trauma	9 (36%)	5 (72%)	0.06
Preexisting corneal disease or surgery	10 (40%)	0	<0.001
Contact lens	4 (16%)	1 (14%)	0.46
Others	1 (4%)	1 (14%)	0.26
Corticosteroid use*	14 (56%)	0	<0.001
Medical treatment success with natamycin and/or amphotericin B, number (%)^†^	5 (33%)	5 (83%)	0.02
Therapeutic surgery, number (%)	8 (32%)	1 (17%)	0.16

*Only the patients with definite history of corticosteroid use were included.

^†^Topical natamycin +/− amphotericin B was used to treat 15 eyes among previous cases and 6 eyes in current series.
